# Short communication: timeline of radiation-induced kidney function loss after stereotactic ablative body radiotherapy of renal cell carcinoma as evaluated by serial ^99m^Tc-DMSA SPECT/CT

**DOI:** 10.1186/s13014-014-0253-z

**Published:** 2014-11-26

**Authors:** Price Jackson, Farshad Foroudi, Daniel Pham, Michael S Hofman, Nicholas Hardcastle, Jason Callahan, Tomas Kron, Shankar Siva

**Affiliations:** Department of Physical Sciences, Peter MacCallum Cancer Centre, East Melbourne, VIC Australia; Department of Radiation Oncology, Peter MacCallum Cancer Centre, East Melbourne, VIC Australia; Sir Peter MacCallum Department of Oncology, University of Melbourne, Parkville, VIC Australia; Department of Radiotherapy, Peter MacCallum Cancer Centre, East Melbourne, VIC Australia; Department of Cancer Imaging, Peter MacCallum Cancer Centre, East Melbourne, VIC Australia; Centre for Medical Radiation Physics, University of Wollongong, Wollongong, NSW Australia

**Keywords:** Stereotactic radiation, Renal cell carcinoma, Primary kidney cancer, Functional imaging, Single photon emission tomography

## Abstract

**Background:**

Stereotactic ablative body radiotherapy (SABR) has been proposed as a definitive treatment for patients with inoperable primary renal cell carcinoma. However, there is little documentation detailing the radiobiological effects of hypofractionated radiation on healthy renal tissue.

**Findings:**

In this study we describe a methodology for assessment of regional change in renal function in response to single fraction SABR of 26 Gy. In a patient with a solitary kidney, detailed follow-up of kidney function post-treatment was determined through 3-dimensional SPECT/CT imaging and ^51^Cr-EDTA measurements. Based on measurements of glomerular filtration rate, renal function declined rapidly by 34% at 3 months, plateaued at 43% loss at 12 months, with minimal further decrease to 49% of baseline by 18 months.

**Conclusions:**

The pattern of renal functional change in ^99m^Tc-DMSA uptake on SPECT/CT imaging correlates with dose delivered. This study demonstrates a dose effect relationship of SABR with loss of kidney function.

## Findings

### Introduction

Stereotactic ablative body radiotherapy (SABR) is an emerging treatment option for inoperable primary renal cell carcinoma (RCC) in both preclinical [1,2] and early clinical trials [3]. Local control rates above 90% have been reported [4-7] and side effects are usually well tolerated [4,8-10]. It is believed that treatment delivered in a single fraction may deliver potent dose effects due to radiation-induced vascular damage, which may prove valuable in the control of radioresistant tumour strains [11]. This effect may have equal consequence in both malignant and healthy tissue; of particular concern for functional renal cells where protracted vascular damage due to ionising radiation may have a significant latent period before manifesting the effects of injury.

There is limited prospective data of renal impairment after SABR to the kidney [3]. Dose–response relationships in this context have been thought to be mitigated by the precision delivery of radiation which may largely spare substantial amounts of renal parenchyma [12,13]. However, no report to date has reported follow-up with accurate functional imaging assessment of both global kidney function using calculated glomerular filtration rate (GFR) and regional kidney function (using DMSA SPECT/CT).

This study presents a preliminary assessment methodology to identify radiation dose effects to renal tissue for a patient receiving single-fraction SABR to a patient with a solitary kidney with primary renal cell carcinoma. Local dose effects to healthy renal tissue are tracked through serial functional SPECT/CT imaging and correlated with radiation dose prescription from treatment planning. This data represents a detailed report of local radiobiological changes following this form of hypofractionated therapy where dose effects are evaluated not just on biochemical markers (GFR & serum creatinine) but through measures of tissue function in 3-dimensional nuclear medicine studies.

### Methodology

**Figure 1:**
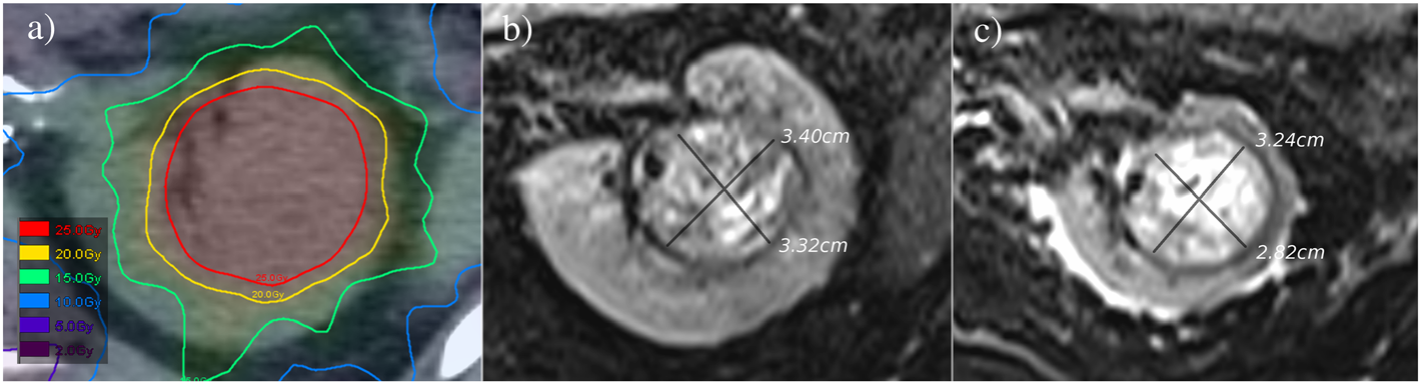
**Axial images of renal carcinoma a) on baseline planning CT, b) pre-therapy T2-weighted MRI, & c) T2 MRI at 18 months post-SABR.**

As part of an independent research board approved prospective clinical trial (clinicaltrials.gov ID NCT01676428), a 42 year old patient was enrolled with an incidentally discovered enlarging primary left renal RCC in a solitary kidney. This patient had been previously treated with orchiectomy, retroperitoneal lymph node dissection and BEP chemotherapy at the age of 19 for a non-seminomatous germ cell tumour. In 2007, he subsequently underwent a right nephrectomy removing two T1a RCCs. In 2012, the new left solitary kidney mass was biopsy confirmed as clear cell carcinoma, measured 38 × 34 × 30 mm on CT imaging, and was situated in the midpole. This patient had renal impairment with an eGFR of 48 ml/min (creatinine 149 μmol/L). The patient was otherwise fit, ECOG performance status of 0, and continued to work full time and exercised daily. A partial nephrectomy was thought not to be technically achievable, and a total nephrectomy would have necessitated immediate dialysis. The lesion was located adjacent to the renal pelvis in contact with renal vessels and ureter, and thought not suitable for radiofrequency ablation. In 2012, he was enrolled as a study participant and underwent a single fraction of SABR (26 Gy) for his solitary left kidney RCC. The radiotherapy plan was generated using a 3D conformal technique with only coplanar beams in the same axial plane in order to avoid beams entering and exiting the superior and inferior poles of the kidney and spare this renal parenchyma (Figure 1). The dose was prescribed to the 80% isodose, ensuring that prescription dose encompassed 99% of the target volume. Radiotherapy was delivered on a Varian Clinac 21iX linear accelerator (Varian Medical Systems, Palo Alto, United States) incorporating vacuum immobilisation with an Elekta BodyFIX device (Elekta Medical Intelligence, Stockholm, Sweden) as previously published [14,15]. Serial nuclear medicine assessments were performed.

### SPECT imaging & glomerular filtration rate

^99m^Tc-DMSA SPECT/CT images (200 MBq injected activity) and GFR by ^51^Cr-EDTA plasma clearance were recorded concurrently at baseline (1 month pre-treatment), and at 2-weeks, 3-, 12-, & 18-months post SABR. Regional renal function was quantified based on measured GFR and total counts in the volume-of-interest comprising the kidney on SPECT imaging. Each voxel’s contribution to total renal function [GFR: (ml/min) per ml tissue] was then calculated based on that scaling factor. The quantified voxel values were compared between baseline and follow-up scans in order to calculate change in local renal function according to prescribed isodose region from the radiotherapy treatment plan.

SPECT images were coregistered to the pre-therapy planning CT and dose prescription image sets. Fused SPECT/CTs were aligned by deformable registration of anatomical, CT volumes with the MIM software package (version 6.1, MIM Software Inc, Cleveland OH, USA). Planning structures were utilised for segmentation of healthy renal tissue [Kidney minus Internal Target Volume (ITV)] [16]. Healthy renal tissue was then differentiated based on radiation isodose zone in 1 Gy increments to record mean counts and volume per zone and identify a dose/response relationship.

### Results

At 18 months post treatment, the patient’s disease is controlled after single-fraction ablative radiotherapy. The positioning of treatment fields within the axial plane permitted the sparing of substantial volumes of renal tissue at the superior and inferior limits of the kidney volume.

**Figure 2:**
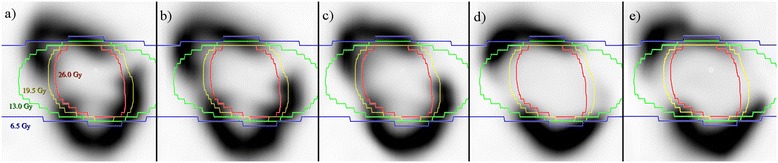
**Serial baseline & post-treatment renal perfusion scans: coronal slices from serial** ^**99m**^ **Tc-DMSA SPECT scans at a) baseline, b) 2 weeks, c) 3 months, d) 12 Months, & e) 18 months.**

**Figure 3:**
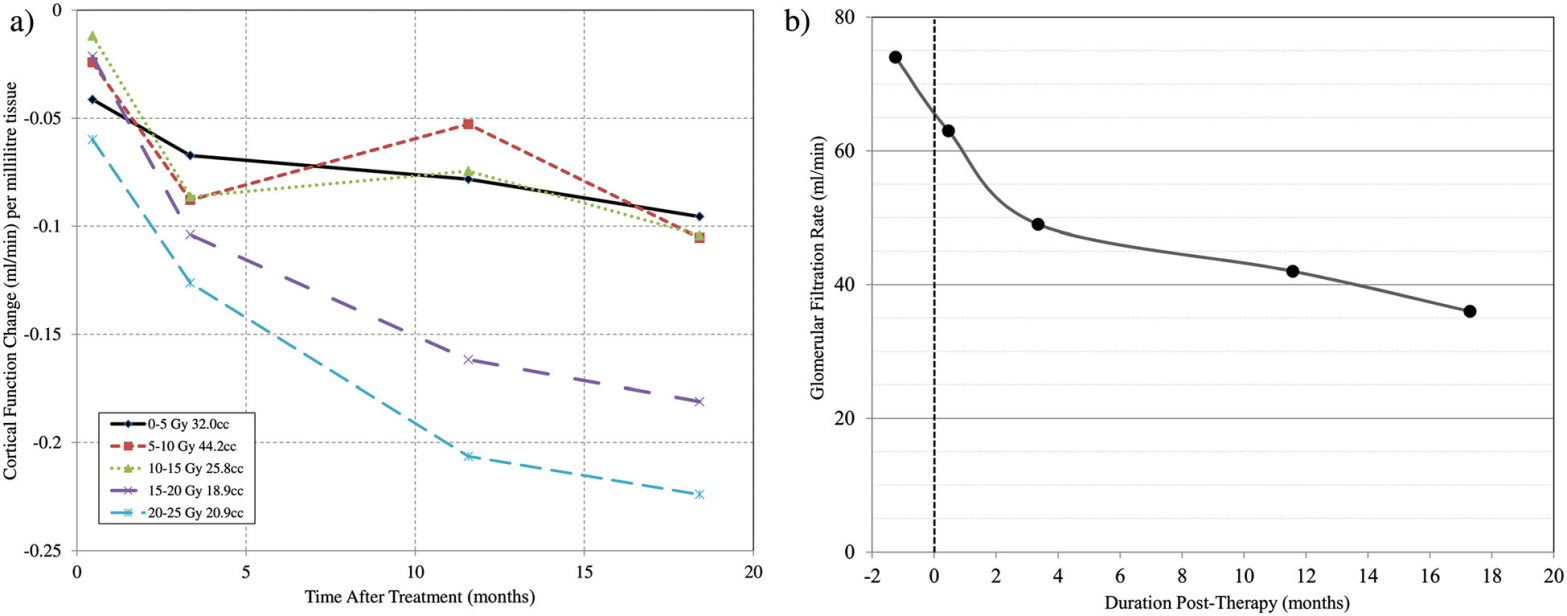
**Temporal renal perfusion change according to delivered radiation dose (a) and measured glomerular filtration rate during the treatment follow-up period (b).**

^99m^Tc-DMSA SPECT/CT scans indicate that renal cortical function is spared to regions with prescribed radiation absorbed dose values below approximately 13 Gy (50% of the prescription dose). In this patient, 60% of tissue was spared appreciable deterioration in regional function, in line with the 13 Gy isodose volume as seen in Figure 2. A modest decline in ^99m^Tc-DMSA uptake is observed at 2-weeks & 3 months post-therapy for high-dose regions. It is apparent from late follow up scans, however, that evidence of radiation induced injury may be delayed for up to a year (Figure 3). Ongoing evolution of dose dependent loss in renal perfusion is demonstrated at 12 months after treatment. At 12 & 18 months the pattern of tracer uptake closely matches the spared dose zones from treatment planning. Minimal decline in perfusion and GFR were observed beyond one year and at a rate that is consistent with the trajectory of progressive chronic kidney disease as previously described by Demirjian et al. [17], (Figure 3b).

From a clinical perspective, the patient continues to work full-time, has an ECOG performance status of 0, and continues to lead a productive lifestyle. He is asymptomatic from his decline in renal function with no significant clinical toxicity.

### Discussion

In this study we find both imaging and biochemical data suggest that dose effects to healthy renal tissue continue to evolve beyond three months after SABR but may stabilise at approximately one year post treatment. In spite of this patient’s previous nephrectomy, platinum-based chemotherapy, and retroperitoneal surgery which contributed to pre-treatment renal dysfunction, the delivery of hypofractionated ablative radiotherapy to the centrally-located tumour in remaining kidney has spared a sufficient volume of functional tissue for the patient to remain free from dialysis.

It is evident that functional nuclear medicine imaging can be informative for assessing normal tissue effects in ablative radiotherapy for renal cell carcinoma. In this instance, DMSA perfusion has been utilised as a surrogate for local renal function. 3-dimensional ^99m^Tc-DMSA SPECT/CT images have indicated that function loss occurs primarily to regions receiving an absorbed dose >13 Gy which corresponds to 50% of the prescription dose and higher in this instance. Whether functional loss within the intermediate to high dose region is clinically consistent across a broader population of patients is yet to be confirmed.

### Conclusion

Single fraction SABR shows promise as viable treatment option for inoperable primary RCC with significant sparing of renal parenchyma in a patient treated with a solitary kidney, although longer-term follow-up is awaited. For the patient reported in this case study, a single-fraction threshold of <13 Gy results in preserved local renal function, with a significant dose/effect relationship at intermediate to high dose ranges (13–25 Gy). This finding requires further validation from multiple patients to confirm the radiation tolerance of renal parenchyma.

## Abbreviations

DMSA: Dimercaptosuccinic acid
ECOG: Eastern Cooperative Oncology Group
EDTA: Ethylenediaminetetraacetic acid
GFR: Glomerular filtration rate
ITV: Internal target volume
RCC: Renal cell carcinoma
SABR: Stereotactic ablative body radiotherapy
